# Successful Surgical Treatment of Mature Teratoma Arising From the Sella

**DOI:** 10.14740/jocmr1998w

**Published:** 2014-11-19

**Authors:** Yaxiong Li, Yuekang Zhang, Jianguo Xu, Ni Chen

**Affiliations:** a Department of Neurosurgery, West China Hospital, Sichuan University, Chengdu, Sichuan Provence, China; b Department of Neurosurgery, The First Hospital of Hebei Medical University, Shijiazhuang, Hebei Provence, China; c Department of Pathology, West China Hospital, Sichuan University, Chengdu, Sichuan Provence, China

**Keywords:** Diagnosis, Craniopharyngioma, Mature teratoma, Radiotherapy, Treatment

## Abstract

Mature teratoma of the pituitary-hypothalamic region is rarely reported in the literature. In this article, we present a 13-year-old girl with clinical and radiological findings that were initially considered as germinoma. However, histological examinations disclosed a mature teratoma. This case highlights that the radiation-induced cerebral edema caused acute hydrocephalus. The mature teratoma is not radiosensitive, and the most appropriate treatment is direct surgery.

## Introduction

Primary intracranial germ cell tumors (GCTs) are relatively rare neoplasms, and account for 0.5% of central nervous system (CNS) tumors [1]. The World Health Organization classified CNS GCTs into eight histological types: germinoma, embryonal carcinoma, yolk-sac tumor, mature teratoma, immature teratoma, teratoma with malignant transformation, choriocarcinoma, and mixed GCTs [2]. The preferential sites of intracranial GCTs are the pineal gland and suprasellar region. Mature teratoma is a sort of GCTs, and the sellar-suprasellar mature teratomas have been rarely reported in the literature.

## Case Report

**Figure 1:**
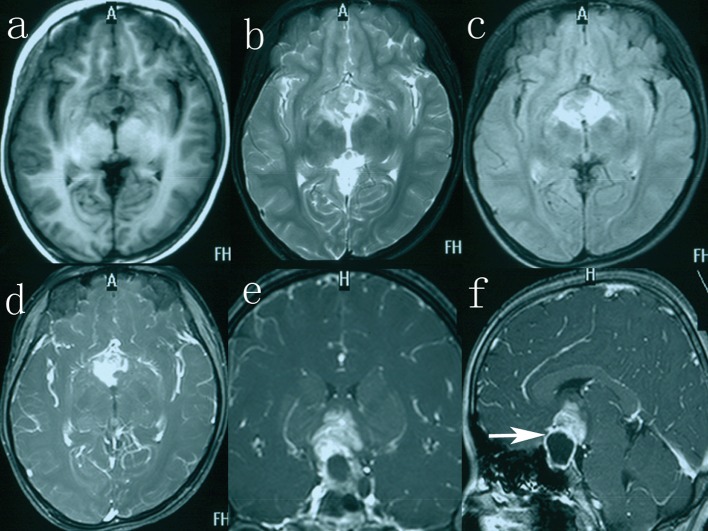
The solid part on axial T1-weighted imaging (a), T2-weighted imaging (b), fluid-attenuated inversion recovery image (c), axial (d) T1-weighted imaging with gadolinium. Coronal (e) and sagittal (f) image, enhanced with gadolinium, showing the tumor consisting of two components, an intrasellar cystic area and a suprasellar solid area (white arrow).

A 13-year-old girl was admitted to our hospital with a 3-year history of polyuria (approximately 3 L of urine every 24 h), polydipsia and amenorrhea. During the 2 months preceding investigation, she complained of blurred vision. In addition, she also had a 1-month history of progressive migraine-like headache. The patient with short stature (height 112 cm) had a weight of 18.5 kg and a bone age of 6 years. Past medical history included myocarditis for 1 year. Physical examination revealed bitemporal hemianopsia, which was more prominent on the right side. The rest of neurological examination was within normal limits. Laboratory evaluation was notable for a urinary specific gravity of 1.003 (normal range (N): 1.010 - 1.025), thyroid-stimulating hormone of 6.830 mU/L (N: 0.27 - 4.2), thyroxine of 38.69 nmol/L (N: 62 - 164), and prolactin of 50 ng/mL (N: 6.0 - 29.9). Seric human chorionic gonadotrophin (HCG-β) and alpha-fetoprotein (AFP) levels were 20 mIU/mL (N < 3.81 mIU/mL) and 50 ng/mL (N < 8 ng/mL), respectively. Growth hormone (GH) level was 1.69 ng/mL (N < 10 ng/mL). Cranial MRI demonstrated a large, sellar and suprasellar, cystic and solid mass extending to the optic chiasm and the right optic nerve (Fig. 1e, f). On T1-weighted images, the cystic portion appeared hypointense with an intensity rim, measuring 1.8 × 2.4 × 2.4 cm in size, and the solid portion showed evidently enhancement after gadolinium-infusion, measuring 2.3 × 2.7 × 2.0 cm in size (Fig. 1a, d). The solid component appeared heterogeneous hyperintense on T2-weighted and fluid-attenuated inversion recovery images (Fig. 1b, c). A presumptive diagnosis of germinoma was made. Then the patient received the radiotherapy (RT) of daily fraction of 1.8 Gy each, 0.9 Gy on each side, to a total of 45 Gy.

**Figure 2:**
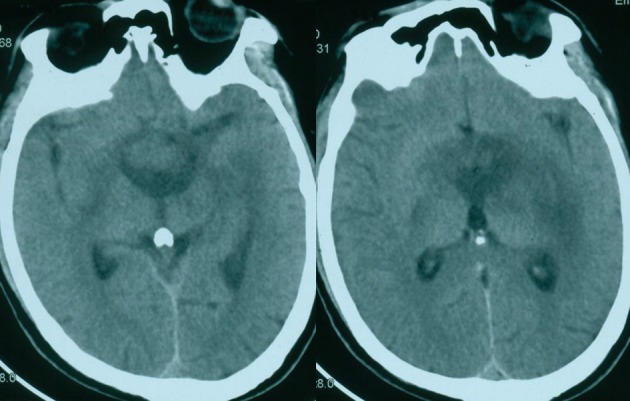
CT showing a suprasellar mass with moderate dilation of the lateral ventricles.

Three days after the RT, however, the patient suffered severe headache, vomiting and visual field defects. Subsequent computed tomography (CT) revealed a mass lesion within the third ventricle accompanied by moderate dilation of the lateral ventricles (Fig. 2). Slight low density developed around the third ventricle on CT. The child was without delay submitted to neurosurgery. Considering the compressive effect and misdiagnosis rate, surgical treatment was planned.

**Figure 3:**
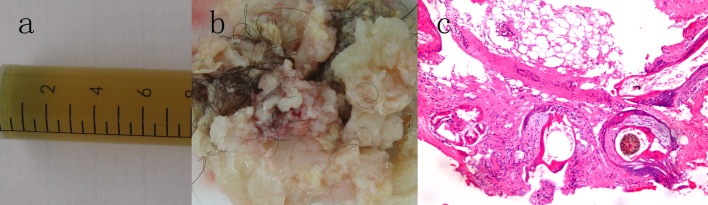
(a, b) Intraoperative photograph revealing the dark yellow cystic fluid and the solid tumor tissue. (c) Photomicrograph of the lesion demonstrating a mature teratoma with adipose tissue, hair, sebaceous glands, and stratified squamous epithelium. Hematoxylin and eosin stain, original magnifications × 100.

The patient underwent a total resection of the mass in the operating room via a right pterional approach. In the operative observation, the tumor consisted of two components, cystic and solid areas. The cystic portion was dark yellow fluid; the solid portion was hair and whitish fat material (Fig. 3a, b). The bone within the tumor was observed intraoperatively. The tumor capsule was progressively separated from both optic nerves and the internal carotid arteries. Histopathology was consistent with mature teratoma comprised of adipose tissue, hair, sebaceous glands, and stratified squamous epithelium (Fig. 2c).

**Figure 4:**
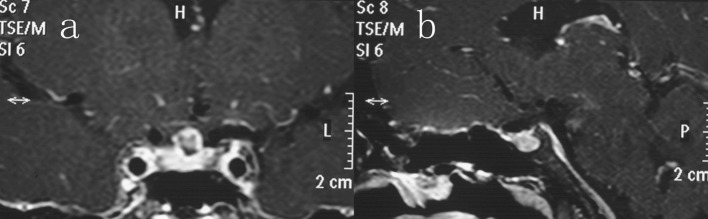
Coronal (a) and sagittal (b) contrast-enhanced imaging after 12 months of follow-up showing complete excision of the sellar teratoma.

Postoperatively, the patient remained stable, although she had transient diabetes insipidus, which resolved by the time of discharge from the hospital. Hormonal replacement was administered. The patient vision improved significantly and she has useful vision in both eyes although the visual field is restricted. No evidence of tumor recurrence was detected on repeat MRI 1 year after surgery (Fig. 4), and the endocrine results were within normal limits.

## Discussion

Mature teratoma is regarded as a benign GCT. The tumor derives from embryonal cells, and has the presence of fully differentiated neuroectodermal, mesodermal and endodermal elements. To our knowledge, there were three cases of primary sellar teratomas in the previous reports [3-5]. Although mature teratomas are usually benign, they tend to adhere firmly to neighboring tissues and rarely become malignant. At present our patient is well, and there is no evidence of recurrent disease, but long-term follow-up is necessary due to malignant transformation and metastase.

The most frequently presenting complaints of suprasellar lesions were hypothalamic-pituitary axis dysfunction such as diabetes insipidus, menstrual irregularities, delayed growth or precocious puberty [6]. GCT, like craniopharyngiomas, may affect the suprasellar region in children leading to the same clinical manifestations. The patient suffered from diabetes insipidus, short stature and amenorrhea because of involvement of the hypothalamus, anterior and posterior pituitary. The mass affected the visual pathway and led to visual disturbances.

Our patient experienced sudden onset of hydrocephalus 3 days after irradiation. CT revealed a large mass of the sellar region with obstructive hydrocephalus (Fig. 2). The mature teratoma grows slowly, not a progression of the tumor, so the radiation effect may be main etiology of the hydrocephalus. The acute radiation-induced cerebral edema should draw attention after RT. It is this that distinguishes their patient from all the previous reports.

MRI plays the most important role in detecting CNS GCTs. Generally, the solid parts of GCTs are isointense with or slightly lower signal than gray matter on T1-weighted imaging, and isotense on T2-weighted imaging [7]. But in our case we found mixed signal of the solid component on T2-weighted MRI (Fig. 1b). There are no specific imaging features, which would enable such a distinction with certainty. The craniopharyngioma and GCT usually share the same radiological features when there is a mature teratoma with calcification.

The histological findings are essential for both craniopharyngioma and GCT diagnosis. In our case, a histopathological verification is made via surgical removal of the lesion. Serum tumoral markers are elevated (HCG-β and AFP), but there was no evidence of secreting cells on immunohistochemical testing. They might have already disappeared after RT.

The best treatment of GCT remains controversial, and includes surgery, RT and chemotherapy. Germinomas are extremely radiosensitive, and the 10-year survival rate of treatment with RT alone is about 90-100% [8]. In the International Society of Paediatric Oncology (SIOP)-CNS-GCT clinical study, histological diagnosis will not be a requirement [9, 10]. According to current scientific literature and the desire of the patient relatives, the child underwent the RT although no histological diagnosis is available. However, our case displayed the worse hydrocephalus symptoms due to the radiation-induced edema. The patient underwent a total resection and was well during the follow-up period of 1 year.

Many kinds of neoplasms including pure germinoma could be treated with irradiation, without surgical resection of the main part of the tumor, but our case underwent open surgery. Surgery brings more benefit than harm to our patient. It could resolve the compressive effect and hydrocephalus, gaining the definite diagnosis. Neurosurgeons should weigh the risks and benefits of surgery to determine the most appropriate plans for each patient. If it is not safe to conduct a surgical removal for patients with sellar masses, when the ventricle is enlarged, endoscopic biopsy of the tumor extending to the third ventricle is rather recommended. Stereotactic biopsy without direct vision for the lesions around the sellar region should be hazardous, because major vessels are located in close proximity to the course of needle insertion. Our study shows that the mature teratoma is not radiosensitive, and the optimum management is direct surgery.

In conclusion, mature teratomas of the pituitary-hypothalamic region are rare. The radiation-induced cerebral edema caused acute hydrocephalus, which is not described in all the previous reports. This should draw attention to the neurosurgeon after irradiation. The clinical features and radiological findings of sellar tumors may be similar in both craniopharyngioma and mature teratoma. Our case suggests that the mature teratoma is not radiosensitive, and the most appropriate treatment is direct surgery.

## Abbreviations

MRI: magnetic resonance imaging
CT: computed tomography
N: normal range
CSF: cerebrospinal fluid
RT: radiotherapy
GCT: germ cell tumor
CNS: central nervous system
